# Amazonian *Phlebovirus* (*Bunyaviridae*) potentiates the infection of *Leishmania* (*Leishmania*) *amazonensis*: Role of the PKR/IFN1/IL-10 axis

**DOI:** 10.1371/journal.pntd.0007500

**Published:** 2019-06-19

**Authors:** Carolina Torturella Rath, Laila Castro Schnellrath, Clarissa R. Damaso, Luciana Barros de Arruda, Pedro Fernando da Costa Vasconcelos, Claudia Gomes, Marcia Dalastra Laurenti, Teresa Cristina Calegari Silva, Áislan de Carvalho Vivarini, Nicolas Fasel, Renata Meirelles Santos Pereira, Ulisses Gazos Lopes

**Affiliations:** 1 Laboratory of Molecular Parasitology, Institute of Biophysics Carlos Chagas Filho, Federal University of Rio de Janeiro, Rio de Janeiro, Brazil; 2 Laboratory of Molecular Biology of Virus, Institute of Biophysics Carlos Chagas Filho, Federal University of Rio de Janeiro, Rio de Janeiro, Brazil; 3 Laboratório de Genética e Imunologia das Infecções Virais, Departamento de Virologia, Instituto de Microbiologia Prof. Paulo de Góes, Universidade Federal do Rio de Janeiro, Rio de Janeiro, Brazil; 4 Department of Arbovirology and hemorrhagic fevers, Instituto Evandro Chagas/SVS/MS, Ananindeua, Brazil; 5 Department of Pathology, Medical School, University of São Paulo, Brazil; 6 Department of Biochemistry, University of Lausanne, Switzerland; 7 Institute of Microbiology Paulo de Góes, Federal University of Rio de Janeiro, Rio de Janeiro, Brazil; Instituto de Ciências Biológicas, Universidade Federal de Minas Gerais, BRAZIL

## Abstract

**Background:**

*Leishmania* parasites are transmitted to vertebrate hosts by phlebotomine sandflies and, in humans, may cause tegumentary or visceral leishmaniasis. The role of PKR (dsRNA activated kinase) and Toll-like receptor 3 (TLR3) activation in the control of *Leishmania* infection highlights the importance of the engagement of RNA sensors, which are usually involved in the antiviral cell response, in the fate of parasitism by *Leishmania*. We tested the hypothesis that *Phlebovirus*, a subgroup of the *Bunyaviridae*, transmitted by sandflies, would interfere with *Leishmania* infection.

**Methodology/Principal findings:**

We tested two *Phlebovirus* isolates, Icoaraci and Pacui, from the rodents *Nectomys* sp. and *Oryzomys sp*., respectively, both natural sylvatic reservoir of *Leishmania (Leishmania) amazonensis* from the Amazon region. Phlebovirus coinfection with *L*. *(L*.*) amazonensis* in murine macrophages led to increased intracellular growth of *L*. *(L*.*) amazonensis*. Further studies with Icoaraci coinfection revealed the requirement of the PKR/IFN1 axis on the exacerbation of the parasite infection. *L*. *(L*.*) amazonensis* and *Phlebovirus* coinfection potentiated PKR activation and synergistically induced the expression of IFNβ and IL-10. Importantly, *in vivo* coinfection of C57BL/6 mice corroborated the *in vitro* data. The exacerbation effect of RNA virus on parasite infection may be specific because coinfection with dengue virus (DENV2) exerted the opposite effect on parasite load.

**Conclusions:**

Altogether, our data suggest that coinfections with specific RNA viruses shared by vectors or reservoirs of *Leishmania* may enhance and sustain the activation of host cellular RNA sensors, resulting in aggravation of the parasite infection. The present work highlights new perspectives for the investigation of antiviral pathways as important modulators of protozoan infections.

## Author summary

Cutaneous Leishmaniasis is highly prevalent worldwide, affecting millions of people in undeveloped countries. *Leishmania* parasites are transmitted to humans by phlebotomine flies. The transmission occurs mainly in rural areas, although the urbanization of the disease is in process. Phlebotomine vectors may also transmit RNA virus (arboviruses) to humans. Our group has previously demonstrated the induction of the host anti-viral pathways by *L*. (*L*.) *amazonensis*, Since some anti-viral response pathways potentiate *L*. (*L*.) *amazonensis* infection and the same group of *Leishmania* arthropod vectors transmits RNA viruses belonging to the genus Phlebovirus, we hypothesized that coinfection could aggravate the parasitism by *L*. (*L*.) *amazonensis*. Using a phlebovirus isolated from *L*. *amazonensis* rodents reservoirs (Icoaraci), we demonstrated that Icoaraci coinfection enhanced the parasite load, both *in vivo* and *in vitro*. These observations were partialy corroborated with Pacui, another Phlebovirus isolate. Increased macrophage infection required IFN type I expression induced by the parasite and amplified during coinfection. Importantly, the infection by another prevalent arbovirus, DENV2, did not result in the exacerbation of the parasite infection. Overall our results support the importance of complex and multifactorial analysis of infectious processes, especially in tropical regions that frequently have endemic areas of overlap.

## Introduction

The World Health Organization acknowledges that leishmaniasis is prevalent in 98 countries and estimates the occurrence of one million new cases of cutaneous leishmaniasis (CL) annually [1]. CL exhibits a broad spectrum of clinical manifestations including ulcerative skin lesions, destructive inflammation of the mucosa (mucocutaneous leishmaniasis), and disseminated and diffuse lesions [2]. *Leishmania (L*.*) amazonensis* is endemic in Brazil and is the causative agent of local CL and anergic diffuse cutaneous leishmaniasis (ADCL), a more severe form of the disease. *Leishmania (L*.*) amazonensis* is accidentally transmitted to humans in the Amazon region by *Lutzomyia flaviscutellata*, since this parasite is naturally found in sylvatic vertebrate reservoirs and its vector [3, 4].

The severity and duration of *Leishmania* infection in humans is shaped by diverse factors ranging from the species of *Leishmania* to the host immune response [5]. More recently, several reports demonstrated the impact of viral endosymbionts on the exacerbation of infection due to *L*. *(Viannia) guyanensis* or *L*. (*V*.*) braziliensis* strains harboring *Leishmania* RNA virus (LRV) [6–8]. According to the current model, LRV-mediated development of exacerbated lesions in vertebrates resulted from aggravation of the inflammatory response by activation of the endosomal double-stranded RNA sensor Toll-like receptor 3 (TLR3) and production of type I interferon and other cytokines [6].

The epidemiological importance and relevance of viral coinfection in the progression of human leishmaniasis is emphasized in HIV1 coinfections studies [9–11]. HIV1-*Leishmania* coinfection is found in several areas in the world and may alter several pathological aspects of visceral and cutaneous leishmaniasis [10,11]. Recently, *in vitro* coinfection study models indicated that other exogenous viruses such as lymphocytic choriomeningitis virus (LCMV) and Toscana virus (TOSV), which are not linked to immunodeficiency, can shape the pathology of *Leishmania* infection via type I interferon (IFN) signaling [12].

Other dsRNA sensors besides TLR3 also play important roles in cell homeostasis and the antiviral response [13] and may engage in inflammatory processes via nuclear factor-κB (NF-κB), Interferon regulatory factor 3 (IRF3) activation and type I IFN expression. The double-stranded RNA-activated kinase (PKR) is an important cytoplasmatic dsRNA sensor. The recognition by PKR of dsRNA intermediates of RNA virus replication promotes PKR dimerization and phosphorylation of the alpha subunit of the eukaryotic initiation factor 2 (eIF2), leading to a partial halt of translation [14–16] and control of some viral infections [17–19]. In addition to the role in the inhibition of protein synthesis, PKR induces type I IFN and IL-10 expression [20] and plays an important role in several cellular processes, such as autophagy [21]. It was shown that *L*. *(L*.*) amazonensis* activates PKR, leading to IL-10, IFNβ and SOD-1 expression, favoring the intracellular growth of *L*. *(L*.*) amazonensis*. Importantly, patients with ADCL exhibit increased expression of PKR and IFNβ [22].

*Leishmania* sandfly vectors may transmit RNA viruses belonging to the genus *Phlebovirus* (*Bunyaviridae*), which are widely distributed throughout Europe, Africa, Central Asia and the Americas [23]. It is conceivable that coinfections between *Leishmania* and phleboviruses may occur because they may share *Leishmania* sandfly vectors and reservoirs [24]. In Brazil, more than 210 different arboviruses (arthropod-borne viruses) have been isolated, of which 200 were in the Brazilian Amazon [25], making the Amazon one of the largest arbovirus reserves in the world of which an important proportion is composed of *Phlebovirus* [26].

The best studied *Phlebovirus* is Rift Valley Fever virus (RVFV), responsible for causing an acute viral illness, which causes fever, most commonly observed in ruminant animals, with the capacity to infect and cause disease in humans [27, 28]. In general, humans infected with *Phlebovirus* may develop a wide range of clinical signs and new emerging Phlebovirus isolates have been described in humans associated to severe acute diseases [29].

In the present work we tested the hypothesis that *Phlebovirus* isolates from the Amazon region enhance *L*. *(L*.*) amazonensis* infection via engagement of the RNA sensor PKR and expression of type I IFN. Our data provide strong evidence that *Phlebovirus* enhances *L*. *(L*.*) amazonensis* infection and may shape the outcome of the pathology.

## Methods

### Cell lines

RAW 264.7 (murine macrophages) and BHK-21 (Baby Hamster Kidney) [C-13] (ATCC: CCL-10) cell lines were cultured in DMEM with high glucose (Gibco) and supplemented with 10% heat-inactivated FBS (Gibco), 100 U/mL penicillin, and 100 g/mL streptomycin at 37°C in a 5% CO_2_ atmosphere. RAW 264.7 cells expressing either PKR K296R, dominant negative PKR (RAW-DN-PKR cells) or an empty vector (RAW-bla cells) were generated as described previously [30].

### Peritoneal macrophages

Thioglycolate-elicited peritoneal macrophages from eight-week-old wild-type (WT) C57BL/6 or interferon-α/β receptor-knockout (IFNAR^-/-^) 129Sv/Ev mice were obtained by injecting 8 mL of PBS into the peritoneal cavity. The cell suspension was washed in PBS one time and then resuspended in serum-free DMEM. Cells were plated on glass coverslips at 2×10^5^/well in 24-well polystyrene plates or 4×10^6^/well in 6-well polystyrene plates and incubated for 1 h at 37°C in a 5% CO_2_ atmosphere. Nonadherent cells were washed out with PBS, and the adherent cell population was incubated for 1 day in DMEM with high glucose and supplemented with 10% heat-inactivated FBS, 100 U/mL penicillin, and 100 g/mL streptomycin for subsequent *Leishmania* or virus infection assays.

### Bone marrow-derived macrophages (BMDM)

Murine bone marrow cells were obtained from 8-week-old WT C57BL/6 mice. Femurs and tibias were disinfected with 70% ethanol, the epiphyses were cut and flushed with a syringe filled with ice-cold DMEM. The cells were centrifuged at 300 × *g*—4°C for 10 min, resuspended in DMEM supplemented with 10% heat-inactivated FBS and 20% L929 supernatant and maintained at 37°C in a 5% CO_2_ atmosphere for 5 days. After this time, BMDM were harvested with ice-cold PBS and cell viability was determined by trypan blue exclusion assay. Next, the cells were plated and infected under the same conditions as peritoneal macrophages.

### *Phlebovirus*: Production, quantification and infection

The *Phlebovirus* Icoaraci (BeAN 24262—ICOV) and Pacuí (BeAN 27326—PACV) used in this work were obtained through Dr. Pedro Vasconcelos (Instituto Evandro Chagas/SVS/MS). Viruses from lyophilized mouse brain macerates were passaged five times in BHK-21 cells to recover infectivity. For viral propagation, BHK-21 cells were seeded in 100-mm plates and the virus inoculum was adsorbed for 2 h at room temperature with a MOI (multiplicity of infection) of 0.01. The inoculum was removed and the cells were maintained in DMEM with high glucose and supplemented with 10% heat-inactivated FBS at 37 °C for 3 days. For viral concentration, cell culture supernatants containing viral particles were collected at 18,000 g for 1 h at 4 °C. The pellet was reconstituted in PBS (Gibco) and stored at -80 °C. Viral titers were determined by plaque assay. BHK-21 cells were seeded in 6-well polystyrene plates (initial inoculum of 1.5×10^6^ cells/well). After 24 h, the cells were infected with a 10-fold of serial dilution of ICOV stock and incubated for 2 h at room temperature. Subsequently, the inoculum was removed and the monolayer covered with semisolid medium composed of DMEM with high glucose supplemented with 10% FBS and 1% methylcellulose (Sigma-Aldrich). Viral plaques were observed 3 days after infection when cells were fixed and stained with 0.1% crystal violet solution containing 10% formaldehyde. Viral plaques were counted and titers were expressed as PFU/mL. For macrophage infections, adsorption was performed for 1 h at 37 °C using a MOI of 1. Infection times varied according to the objective of the experiment.

### Dengue 2: Production, quantification and infection

Dengue serotype 2 (16681 strain) was propagated in the C6/36 cell line (ATCC-CLR1660), maintained at 28 °C in Leibovitz (L-15) medium (Life Technologies) supplemented with 0.2% of L-glutamine, 10% of tryptose phosphate broth (Sigma), 0.75 g/L sodium bicarbonate (Sigma), 10% FBS (Life Technologies). C6/36 cells were infected with a MOI of 0.1. Nine days postinfection, the supernatants were harvested, centrifuged at 400g for 10 min and filtered. Supernatants were stored at -80 °C. Viral titers were determined by plaque assay performed on BHK-21 cells [31]. Infections in peritoneal macrophages followed the same conditions performed with ICOV and PACV, however using a MOI of 2.

### Parasites: Culture conditions and infection

*L*. *(L*.*) amazonensis* (WHOM/BR/75/Josefa) promastigotes were grown at 26 °C in Schneider’s insect medium (Sigma-Aldrich) supplemented with 10% FBS, 100 U/mL penicillin and 100 g/mL streptomycin. Promastigotes from 4- to 5-day stationary cultures were used for experiments throughout. Cells were infected for 1 h at 35 °C with *Leishmania* stationary-phase promastigotes with a parasite:cell ratio of 5:1. Noninternalized promastigotes were washed out, fresh medium was added, and cultures were maintained at 35 °C in a 5% CO_2_ atmosphere for various periods of time. In some experiments, cells were pretreated with 300 nM of the PKR inhibitor CAS-608512-97-6 (Millipore) or infected with ICOV before *Leishmania* infection. Infected macrophages were counted by light microscopy to assess the infection index as follows: 100 Giemsa-stained cells were inspected and the percentage of infected macrophages was multiplied by the average number of amastigotes per macrophage.

### *In vivo* coinfection

The cushion footpads of C57BL/6 (WT) mice were subcutaneously injected with 5×10^6^ *L*. *(L*.*) amazonensis* stationary-phase promastigotes and 2.25×10^5^ PFU of ICOV virus in PBS. The progression of lesions was measured weekly with a digital caliper and expressed as the difference between the thickness of the infected and noninfected footpads. At the indicated time-points, the mice were euthanized for immunohistochemical analysis.

### Histopathological characterization and immunohistochemistry quantitative analysis of IFNβ-expressing cells and parasite density in infected footpads of mice

Immunohistochemical analysis was used to assess the densities of IFNβ-, IL-10- and iNOS-expressing cells and of *L*. *(L*.*) amazonensis* amastigotes in histological sections prepared from footpad biopsies of mice infected singly or coinfected with *L*. *(L*.*) amazonensis* and ICOV. The biopsies were fixed in 10% buffered formalin, embedded in paraffin, and sectioned at 4 μm. The sections were stained with hematoxylin and eosin (H&E) to evaluate the histopathological changes in the skin lesions of different experimental groups. Tissue sections were stained with anti-*Leishmania* primary antibody (mouse hyperimmune serum, LIM-50/HCFMUSP), antibodies specific for IFNβ (SC-20107, Santa Cruz Biotechnology), IL-10 (SC-73309, Santa Cruz Biotechnology) and iNOS (N20, SC-651, Santa Cruz Biotechnology). To evaluate the amastigote, IFNβ^+^, IL-10^+^ and iNOS^+^ cells density, ten fields for each histological section were photographed with a 40X objective, and the number of immunostained amastigotes was quantified using Axiovision 4.0 software on a computer coupled to an optical microscope (Axioskop 2 plus; Carl-Zeiss, Jena, Germany). Subsequently, the density of immunostained parasites was calculated by determining the average number of amastigotes per mm^2^ [32]. Quantitative analysis of reactive cells positive for IFNβ, IL-10 and iNOS was achieved through the processing of selected optical fields by Axioplan 2 Plus software (Carl Zeiss, Oberkochen, Germany). The density of parasites and positive cells was calculated by determining the average number of amastigotes and cells per square micrometer examined.

### Immunoblotting

Peritoneal macrophages (4×10^6^) were washed twice with ice-cold PBS and then lysed in 80 μL of lysis buffer (50 mM Tris-HCl, pH 7.5; 5 mM EDTA; 10 mM EGTA; 50 mM NaF; 20 mM glycerophosphate; 250 mM NaCl; 0.1% Triton X-100; and 1 g/mL BSA) to which a 1:100 dilution of protease inhibitor cocktail (Sigma-Aldrich) and a 1:50 dilution of phosphatase inhibitor cocktail (Sigma-Aldrich) was added. Proteins were subjected to electrophoresis in 10% SDS-polyacrylamide gels and transferred to a PVDF membrane (Bio-Rad). The following primary antibodies were used in this study: PKR (SC-708, Santa Cruz Biotechnology), phospho-PKR (07–886, Millipore), eIF2α and phospho-eIF2α (9722 and 9721, respectively, Cell Signaling Technology), Ak1 and phospho-Akt1 (9272 and 9271, respectively, Cell Signaling), and β-actin (A00702-SZ, GenScript), followed by goat anti-rabbit or goat anti-mouse horseradish peroxidase-conjugated IgG (sc-2030 and sc-2005, respectively, Santa Cruz Biotechnology). The proteins were detected by the Pierce ECL Western Blotting Substrate (Thermo Fisher Scientific) and bands were quantified by densitometry using Adobe Photoshop CS6.

### Semiquantitative RT-PCR

Peritoneal macrophages were harvested, and total RNA was obtained with the Direct-zol RNA MiniPrep Plus kit (Zymo). First-strand cDNA synthesis was performed in a reaction containing Improm-II Reverse Transcriptase (Promega), a mix of dNTPs, and random primers (Promega), as described by the manufacturer. RT-PCR was performed using primers for the N protein gene of ICOV (sense 5’-AGGTGAGGCTGTAAATCTTG-3’ and antisense 5’-TCACATCATCCTTCCAAGTG-3’) or for the GAPDH gene (sense 5’-TTGACCAACTGCTTAGC-3’ and antisense: 5’-GGCATGGACTGTGGTCATGAG-3’), 2.5 U of GoTaq DNA polymerase (Promega), and 1.5 mM MgCl_2_ in an appropriate buffer at an annealing temperature of 48 °C and 40 amplification cycles. PCR products were separated on a 1.2% agarose gel, stained with ethidium bromide, and photographed in a UV transilluminator.

### Real-time quantitative RT-PCR (qRT-PCR)

Total RNA from peritoneal macrophages (4×10^6^) was extracted via the Direct-zol RNA MiniPrep Plus kit (Zymo). RNA (1 μg) was reverse transcribed to first-strand cDNA with ImProm-II (Promega) and oligo(dT) 12–18 primer, according to the manufacturer’s instructions. Real-time PCR was performed using primers for the IFNβ (sense 5’ TCC AAG AAA GGA CGA ACA TTC G 3’ and antisense 5’ TGA GGA CAT CTC CCA CGT CAA 3’), IL-10 (sense 5’ CCC AGA AAT CAA GGA GCA TT 3’ and antisense: 5’ TCA CTC TTC ACC TGC TCC AC 3’) and GAPDH gene (sense 5’ TTG ACC AAC TGC TTA GC 3’ and antisense 5’ GGC ATG GAC TGT GGT CAT GAG 3’). Amplicon specificities were compared by the presence of a single melting temperature dissociation curve after real-time RT-PCR runs. Real-time qRT-PCR was performed in the Applied Biosystems StepOne detection system (Applied Biosystems) using GoTaq qPCR Master Mix (Promega). All qRT-PCR experiments were performed 3 times, and the experimental qRT-PCR data from the experiments were normalized using GAPDH primers as an endogenous control. All expression ratios were determined by the relative gene expression ΔΔCt method using StepOne 2.0 software 2.0 (Applied Biosystems).

### Cell viability assay

Peritoneal macrophages of WT mice were seeded in a 24-well plate. After 24 h, cells were infected with ICOV (MOI of 1) for 1 h at 37 °C. Then, cells were infected for 1 h at 35 °C with *Leishmania* stationary-phase promastigotes with a parasite:cell ratio of 5:1. Noninternalized promastigotes were washed out, fresh medium was added, and cultures were maintained at 35 °C in a 5% CO_2_ atmosphere for 24 h and 48 h. The culture medium was removed and 100 μL of MTT (3-(4,5-dimethylthiazol-2-yl)-2,5-diphenyltetrazolium bromide) (1 mg/mL) was added. After 1 h of incubation at 37 °C, MTT was removed and 250 mL of isopropyl alcohol was added. Samples were homogenized and optical density was measured at 570 nm.

### Statistical analysis

Data were submitted to Shapiro-Wilk test for normal distribution and, further, analyzed by two-way ANOVA for independent samples followed by a post hoc Dunnett’s test (comparing to a single control group) or a Tukey’s test (with no designated control group), using GraphPad Prism 5 software (San Diego, CA, USA). Data were presented as the mean values ± standard error of the mean (SEM) of three independent experiments. Comparisons between means were considered to be statistically significant when *p* < 0.05.

### Ethics statement

The methods carried out in this work are in accordance with the guidelines approved by the Ethical Committee of biological Research Experimentation, Federal University of Rio de Janeiro, Brazil. All the experimental protocols were approved by Federal University of Rio de Janeiro Committee for Animal Use (permit numbers: IMPPG 024 and IBCCF171.

## Results

### Intracellular growth of *Leishmania (L*.*) amazonensis* is exacerbated in response to coinfection with *Phlebovirus*

**Fig 1:**
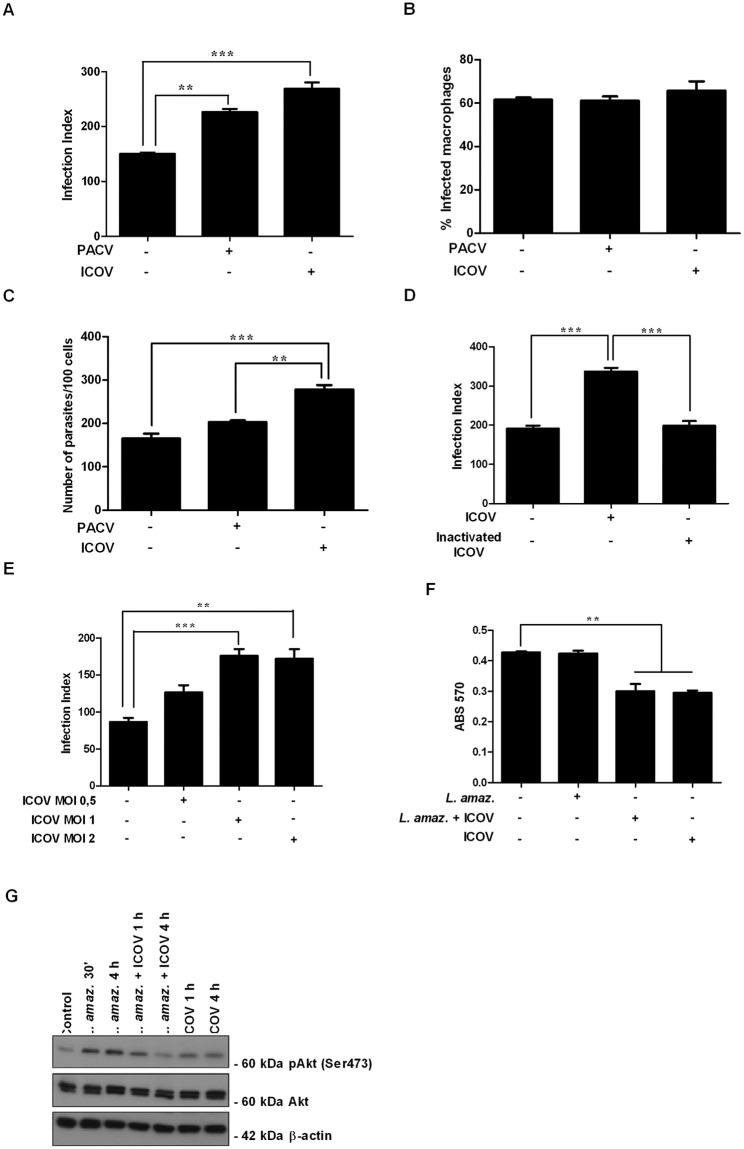
Coinfection with Amazonian *Phlebovirus* potentiates *Leishmania (L*.*) amazonensis* infection *in vitro*. (A—D) Peritoneal macrophages from wild-type C57BL/6 mice were infected with Pacuí (BeAN 27326) or Icoaraci (BeAN 24262) virus for 1 h, followed by infection with stationary-phase promastigotes of *L*. *(L*.*) amazonensis* at a ratio of 5 parasites/cell. At 48 h postinfection, one hundred Giemsa-stained cells were inspected, and the infection index was calculated (percentage of infected macrophages multiplied by average number of amastigotes per macrophage). (B) The percentages of infected macrophages and (C) the number of parasites/100 cells were evaluated. (E) Ultraviolet light-inactivated Icoaraci viral particles were also tested in the coinfection model. The viability of virus-infected or *Leishmania* coinfected macrophages was tested by (F) MTT assay after 24 h and 48 h postinfection and (G) phosphorylation for Akt after 30 min, 1 h or 4 h. The western blot images are representative of three independent experiments. Asterisks indicate significant differences between groups by ANOVA, with * *p* <0.05; ** *p* <0.0017; *** *p* <0.0001 (n = 3).

Because the classical antiviral response mediated by the PKR/IFN1 axis is triggered by *L*. *(L*.*) amazonensis* and further dsRNA or type I IFN addition to infected cells resulted in enhancement of the parasite growth, we predicted that coinfections with arboviruses may lead to sustained PKR expression and enhanced expression of IFNβ [21]. To address the hypothesis that virus coinfection could increase the *L*. *(L*.*) amazonensis* load, macrophages were coinfected with *L*. *(L*.*) amazonensis* and Icoaraci or Pacuí phleboviruses for 48 h, and the parasite load was measured. The experimental model comprised coinfection with *Phlebovirus* isolated from wild rodent, which are reservoirs of *Leishmania* species in Brazil. Our results indicate that *Phlebovirus* coinfection promoted a higher infection index compared to single *L*. *(L*.*) amazonensis* infection (Fig 1A). The coinfection did not alter the percentage of infected macrophages (Fig 1B) but did increase the total number of parasites in ICOV coinfections compared to macrophages infected with only *L*. *(L*.*) amazonensis* or *L*. *(L*.*) amazonensis* and PACV (Fig 1C). The levels of viral nucleoprotein synthesized in the infected macrophages were evaluated to confirm the infection of macrophages by ICOV (Suplementary Fig 1A). The viability of the viral particles released by macrophages were also evaluated (Suplementary Fig 1B). Infectious viral particles are capable of lysing cell monolayer, forming viral plaques. Thus, after 3 days in culture, we observed plaque formation in the BHK-21 monolayer, indicating the infectivity of the viral particles produced by macrophages. Considering that Icoaraci viral production was more amenable than Pacui, we decided to continue the coinfection study solely with Icoaraci virus.

Importantly, UV-inactivated Icoaraci viral particles did not exacerbate *L*. *(L*.*) amazonensis* infection (Fig 1D). To verify the optimal viral MOI for enhancement of *L*. *(L*.*) amazonensis* infection, peritoneal macrophages were infected with four different viral MOIs (Fig 1E). The MOIs of 1 and 2 led to increased parasite load, but we did not observe significant differences between MOIs of 1 and 2. Therefore, we chose the MOI of 1 to proceed with the next tests. To address whether *Phlebovirus* infection would impact macrophage viability, we carried out MTT assays 24 h postinfection (Fig 1F). Accordingly, it was observed that Icoaraci virus reduces Akt phosphorylation induced by *L*. *(L*.*) amazonensis* 1 h and 4 h postinfection (Fig 1G).

### *Phlebovirus* (Icoaraci) infection activates PKR signaling and enhances *L*. *(L*.*) amazonensis* infection

**Fig 2:**
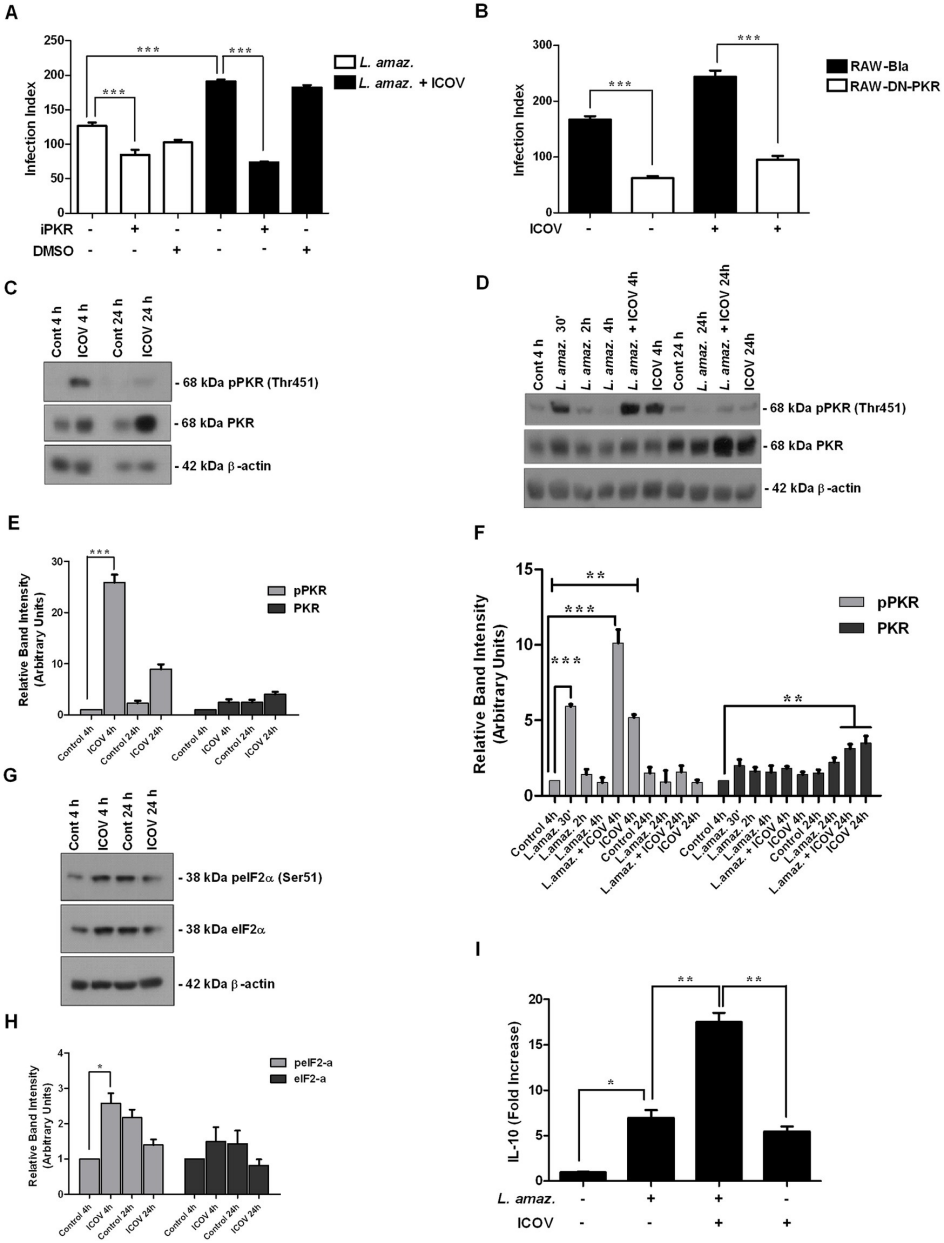
The double-stranded RNA sensor PKR is activated in response to Icoaraci infection and is required for the potentiation of *Leishmania (L*.*) amazonensis* infection. Peritoneal macrophages from wild-type C57BL/6 pretreated with PKR inhibitor CAS-608512-97-6 (A) or RAW-bla and RAW-DN-PKR cells (B) were infected with Icoaraci virus at a MOI of 1 for 1 h, followed by infection with stationary-phase promastigotes of *L*. *(L*.*) amazonensis* at a ratio of 5 parasites/cell for 48 h. One hundred Giemsa-stained cells were inspected, and the infection index was calculated (percentage of infected macrophages multiplied by average number of amastigotes per macrophage). (C—G) Protein extracts from Icoaraci-infected, (D) *L*. *(L*.*) amazonensis* or coinfected macrophages were submitted to Western Blot analysis using anti-phosphoPKR, anti-PKR, anti-phosphoeIF2-α, anti-eIF2-α or anti-β-actin (endogenous control) antibodies, as depicted in the figure. Densitometric analysis of the blots for phosphor-PKR and total PKR (E—F) or phospho-eIF2-α and eIF2-α total (H) was performed. (I) qPCR analysis for IL-10 expression was performed with the RNA obtained 4 h after *L*. *(L*.*) amazonensis-*, Icoaraci- or Icoaraci/*L*. *(L*.*) amazonensis-*infection. The western blot images are representative of three independent experiments. Asterisks indicate significant differences between groups by ANOVA, with * *p* <0.05; ** *p* <0.0017; *** *p* <0.0001 (n = 3).

Because PKR plays a key role in *L*. *(L*.*) amazonensis* infection [20, 22], we decided to test whether PKR was activated and required for the enhancement of parasite growth due to *Phlebovirus* coinfection. Pharmacological inhibition of PKR reduced the enhanced infection promoted by ICOV coinfection (Fig 2A). This result was further corroborated in coinfected DN-PKR (dominant-negative PKR) macrophages (Fig 2B). These results show that PKR activation is required for the enhancement of *L*. *(L*.*) amazonensis* infection in the *Phlebovirus* coinfection model.

The NSs protein (non-structural protein encoded in the S-segment), is considered the main virulence factor for Bunyavirus, and it plays an important role in evasion of innate immunity of the host. It has been reported that NSs of RVFV induces the degradation of PKR via the proteasome as an evasion of the host antiviral response [33, 34]. We decided to investigate whether Icoaraci infection would modulate PKR signaling. Western blot assays show that Icoaraci infection, in fact, activates PKR in macrophages after 4 h of infection. In addition, PKR levels were increased both at 4 h and at 24 h postinfection (Fig 2C), as shown by densitometry (Fig 2E), indicating that, unlike RVFV and TOSV and similar to Sandfly Fever Sicilian virus (SFSV), ICOV is not able to degrade PKR. Importantly, the infection by *L*. *(L*.*) amazonensis* induced PKR phosphorylation in macrophages 30 minutes postinfection (Fig 2D). This induction was inversely proportional to the time of infection. However, 4 h after coinfection, we observed the rescue of phosphorylated PKR levels induced in response to ICOV. Moreover, total PKR levels were increased in response to coinfection after 24 h, indicating a possible synergism between *L*. *(L*.*) amazonensis* and ICOV, as shown by densitometry (Fig 2F). The activation of PKR was confirmed by the detection of phosphorylated eIF2-α, a well-characterized PKR substrate (Fig 2G). The increase in eIF2-α levels was observed only 4 h after infection and was not altered 24 h postinfection, as shown by densitometry (Fig 2H). These results indicate that ICOV induces and sustains PKR activation during coinfection with *L*. *(L*.*) amazonensis*.

Interleukin-10 expression is induced due to PKR activation and is associated with diminished leishmanicidal mechanisms in infected macrophages [20]. Thus, we decided to investigate whether the exacerbation of *L*. *(L*.*) amazonensis* infection promoted by ICOV was associated with increased IL-10 expression. Fig 2I shows that IL-10 expression was enhanced in the coinfection condition compared to single infection by *Leishmania* or ICOV.

Altogether, this set of results support the notion that ICOV sustains PKR activation, leading to high levels of IL-10 and indicating possible synergism between *L*. *(L*.*) amazonensis* and ICOV.

### Icoaraci virus infection activates the type I IFN signaling pathway, favoring *L*. *(L*.*) amazonensis* infection

**Fig 3:**
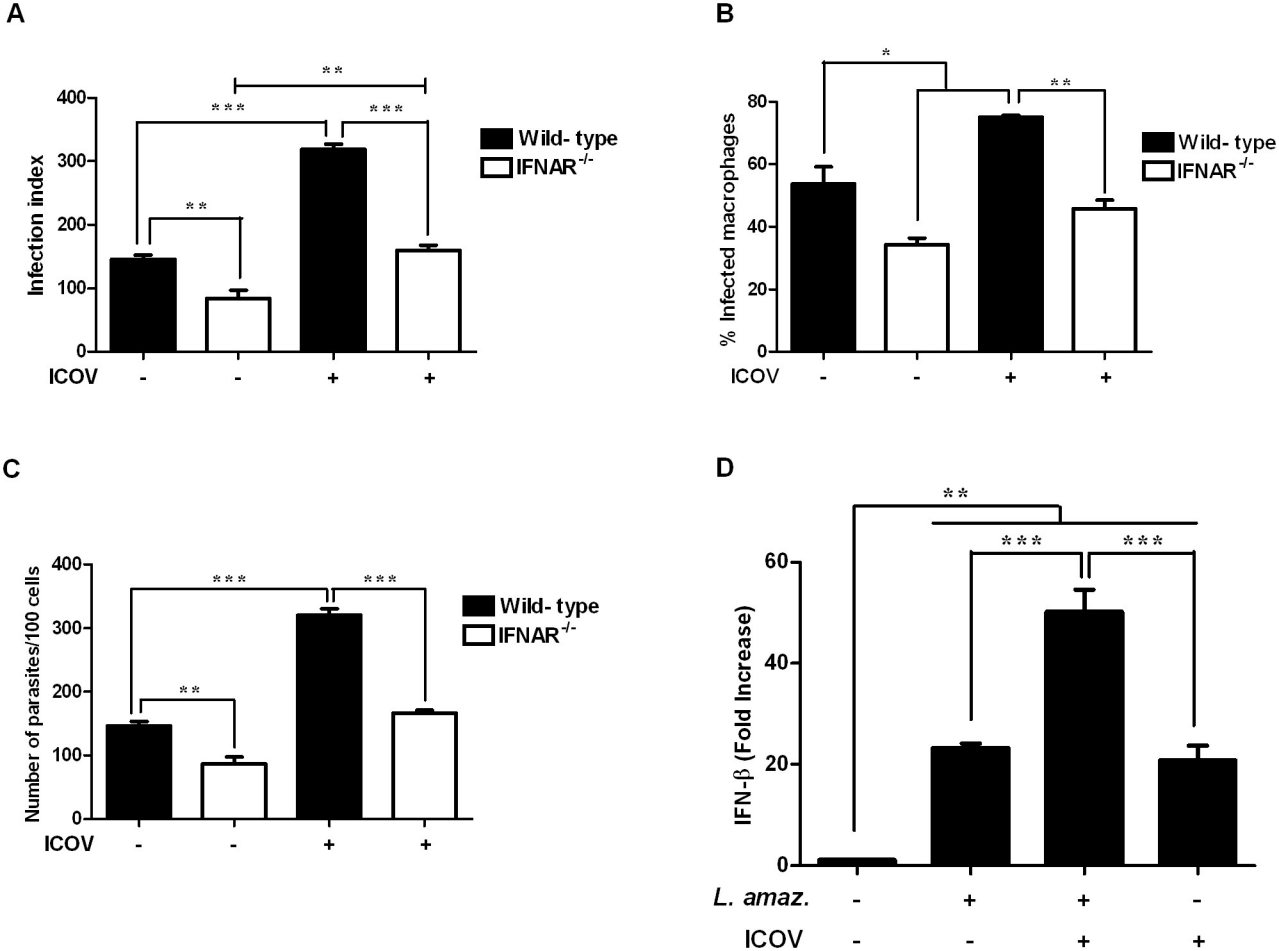
The Type I IFN signaling pathway is required for the enhancement of Icoaraci virus-induced infection. Peritoneal macrophages from wild-type 129Sv/Ev or IFNAR^-/-^ 129Sv/Ev mice were infected with Icoaraci virus at a MOI of 1 for 1 h, followed by infection with *L*. *(L*.*) amazonensis* promastigotes at a ratio of 5 parasites/cell for 48 h. (A) One hundred Giemsa-stained cells were inspected, and the infection index was calculated. (B) The percentages of infected macrophages and (C) the number of parasites/100 cells were evaluated. (D) IFNβ expression analysis was performed by quantitative real-time PCR on RNA isolated 4 h postinfection (*Leishmania*-infected or Icoaraci/*Leishmania*-coinfected) from peritoneal C57BL/6 macrophages. Asterisks indicate significant differences between groups by ANOVA, with * *p* <0.05; ** *p* <0.0017; *** *p* <0.0001 (n = 3).

The importance of IFNβ in exacerbating ICOV-induced *L*. *(L*.*) amazonensis* infection was analyzed using macrophages from 129Sv/Ev mice and knockouts for the type I IFN receptor (IFNAR^-/-^). As expected, we observed a significant reduction in the infection index of *L*. *(L*.*) amazonensis* in IFNAR^-/-^ macrophages compared to WT macrophages. The exacerbation of *L*. *(L*.*) amazonensis* infection by ICOV was also reduced by the absence of the IFNA receptor (Fig 3A). In addition, we observed that both the percentage of infected macrophages (Fig 3B) and the increase in the number of parasites per cell (Fig 3C) were dependent on the type I IFN pathway. We observed increased infection index during coinfection when compared to *L*. *(L*.*) amazonensis* infection (S2A Fig). The percentage of infected macrophage (S2B Fig) and the number of parasites per cell (S2C Fig) were also increased in response to ICOV infection.

Importantly, macrophages infected with ICOV also produced increased levels of IFNβ. Remarkably, in coinfected macrophages, the expression of IFNβ was even greater when compared to individual infections (Fig 3D). As expected, the coinfection of BMDM also led the up-regulation of IFNβ (S2D Fig).

These data indicate a possible synergism between *L*. *(L*.*) amazonensis* and ICOV in promoting the expression of this cytokine, which may be related to the increase in the intracellular proliferation of *Leishmania*.

### Parasite burden, lesion progression and IFNβ expression are increased in *in vivo* coinfection assays

**Fig 4:**
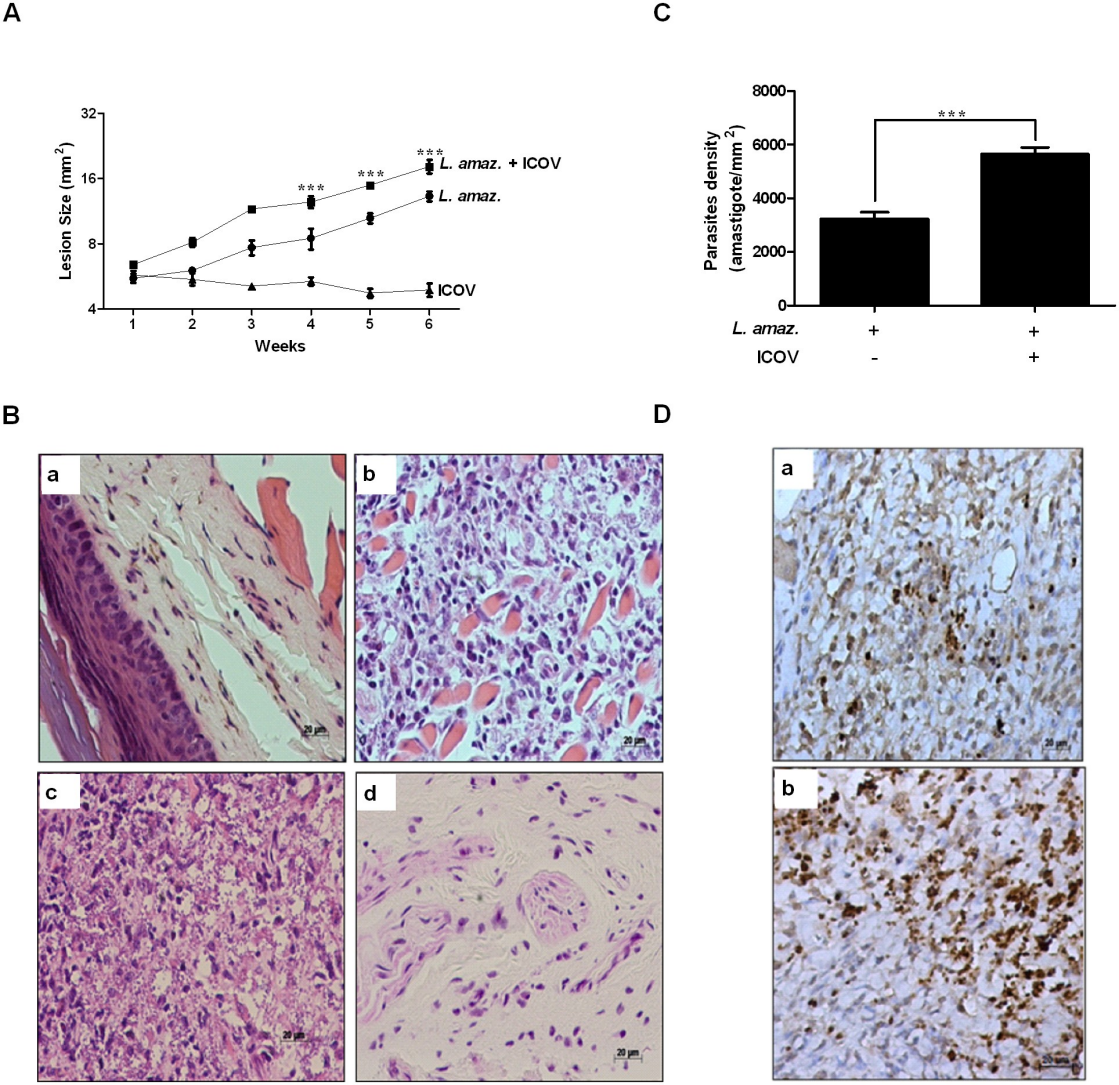
Coinfection of mice with Icoaraci exacerbates the infection of *Leishmania (L*.*) amazonensis*. (A) Wild-type C57BL/6 mice were infected with stationary-phase promastigotes of *L*. *(L*.*) amazonensis* at a ratio of 5 x 10^6^, 2,25 x 10^5^ PFU of Icoaraci virus, or coinfected. The diameter of the paw was evaluated once a week and expressed as the difference between the size of the infected and uninfected paws. (B) In the fifth week after infection, a histopathological analysis of skin lesions of mouse footpads was performed (a—without infection, b—*L*. *(L*.*) amazonensis* infection, c—*L*. *(L*.*) amazonensis* + ICOV infection and d—ICOV infection). (C) Graphical analysis of reactive cells positive for *Leishmania*. (D) The density of amastigotes was evaluated by immunohistochemical reaction with antibody against *Leishmania* (a—*L*. *(L*.*) amazonensis* infection, b—*L*. *(L*.*) amazonensis* + ICOV infection). Images are representative of 5 animals in different groups. Asterisks indicate significant differences between groups by ANOVA or Student’s *t*-test, with *** *p* <0.0001.

**Fig 5:**
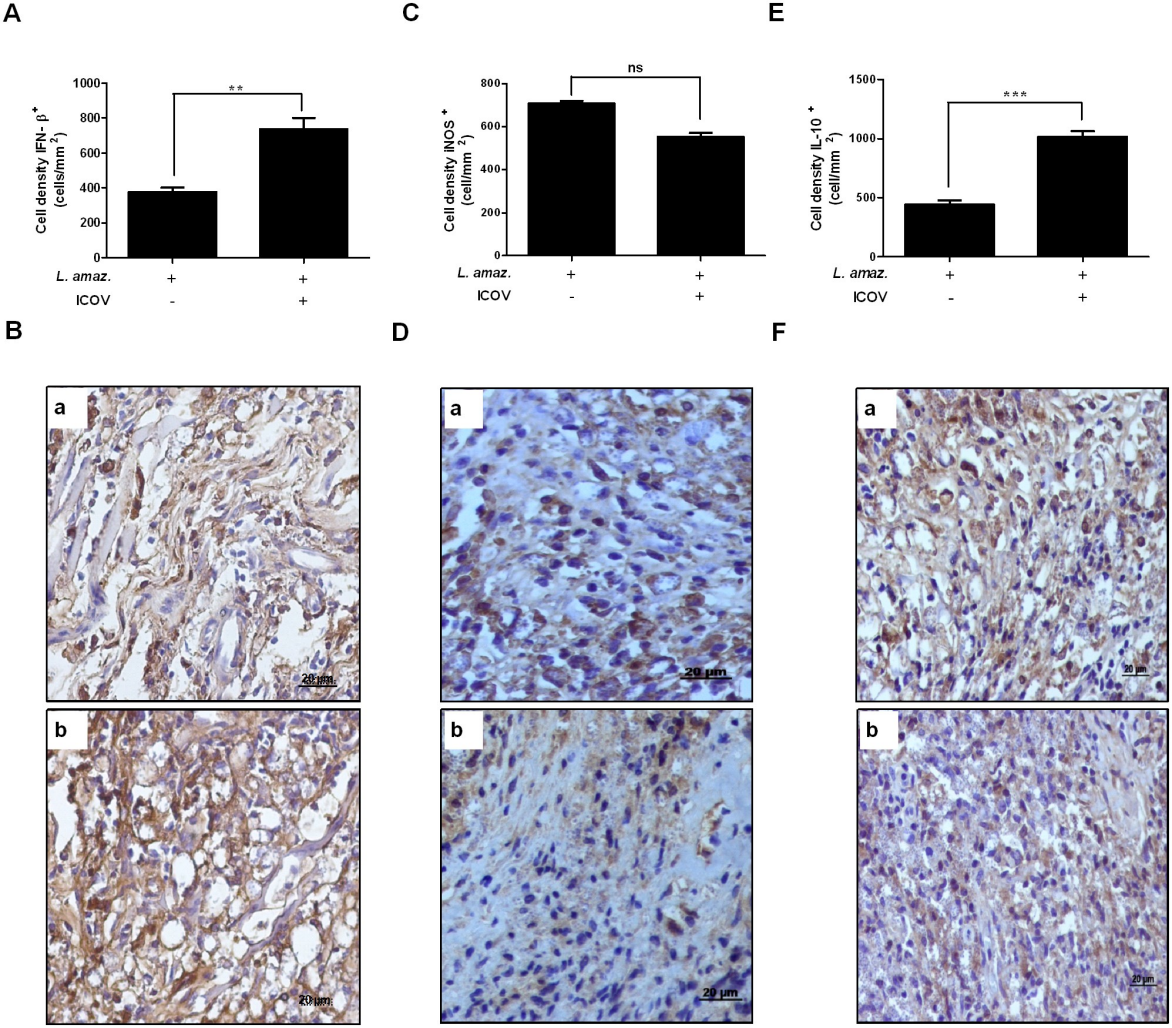
The favoring infection of *L*. *(L*.*) amazonensis* during coinfection with Icoaraci virus is due to increased expression of IFNβ and IL-10. Wild-type C57BL/6 mice were infected with stationary-phase promastigotes of *L*. *(L*.*) amazonensis* at a ratio of 5 x 10^6^, 2,25 x 10^5^ PFU of Icoaraci virus, or coinfected. Graphical analysis of reactive cells positive for IFNβ (A), iNOS (C) and IL-10 (E). The density of IFNβ-, IL-10- and iNOS-expressing cells was evaluated by immunohistochemical reaction with antibody against IFNβ (B: a—*L*. *(L*.*) amazonensis* infection, b—*L*. *(L*.*) amazonensis* + ICOV infection), iNOS (D: a—*L*. *(L*.*) amazonensis* infection, b—*L*. *(L*.*) amazonensis* + ICOV infection) and IL-10 (F: a—*L*. *(L*.*) amazonensis* infection, b—*L*. *(L*.*) amazonensis* + ICOV infection). Images are representative of 5 animals in different groups. Asterisks indicate significant differences between groups by Student’s *t*-test, with ns: not significant; ** *p* <0.0058; *** *p* <0.0001.

To validate our *in vitro* observations, we infected mice with 5×10^6^ stationary-phase promastigotes of *L*. *(L*.*) amazonensis*, 2.25×10^5^ PFU of ICOV or coinfection of both in the footpad. Analysis of the lesions indicated a significant size increase in the coinfected animals compared to the animals solely infected with *L*. *(L*.*) amazonensis* in the fourth week after infection. In the animals infected only with ICOV, we did not observe any lesion (Fig 4A). Histopathological analyses of the footpads of the animals infected with *L*. *(L*.*) amazonensis* showed apparent inflammatory infiltrates (Fig 4B-b). In the animals infected with ICOV (Fig 4B-d), we observed a discrete inflammatory infiltrate close to the normal aspect (similar to that observed in the animals without infection, shown by the Fig 4B-a), whereas the coinfected animals showed an intense inflammatory infiltrate (Fig 4B-c). As predicted, we observed a significant increase in parasite load in the tissues of coinfected animals (Fig 4D-b) when compared to animals infected only with *L*. *(L*.*) amazonensis* (Fig 4D-a). Parasites density by macrophages infected and coinfected were plotted on the graph (Fig 4C). We also compared the expression of IFNβ (Fig 5B), iNOS (Fig 5D) and IL-10 (Fig 5F) in tissue samples obtained from infected mice with *L*. *(L*.*) amazonensis* and coinfected with *L*. *(L*.*) amazonensis* and ICOV. We observed a significant increase in IFNβ- and IL-10 positive cells (Fig 5B-b and 5F-b) in the tissues of coinfected animals when compared to animals infected only with *L*. *(L*.*) amazonensis* (Fig 5B-a and 5F-a). The number of iNOS expressing cells were same between samples from infected (Fig 5D-a) and coinfected animals (Fig 5D-b). The expression of IFNβ (Fig 5A), iNOS (Fig 5C) and IL-10 (Fig 5E) by macrophages was plotted on the graph. These data corroborate our previous *in vitro* observations and underline the importance of *Phlebovirus* and of the increase of IFNβ and Il-10 expression in aggravation of *in vivo Leishmania* infection.

### Dengue virus 2 (DENV2)coinfection does not enhance *L*. *(L*.*) amazonensis* load

**Fig 6:**
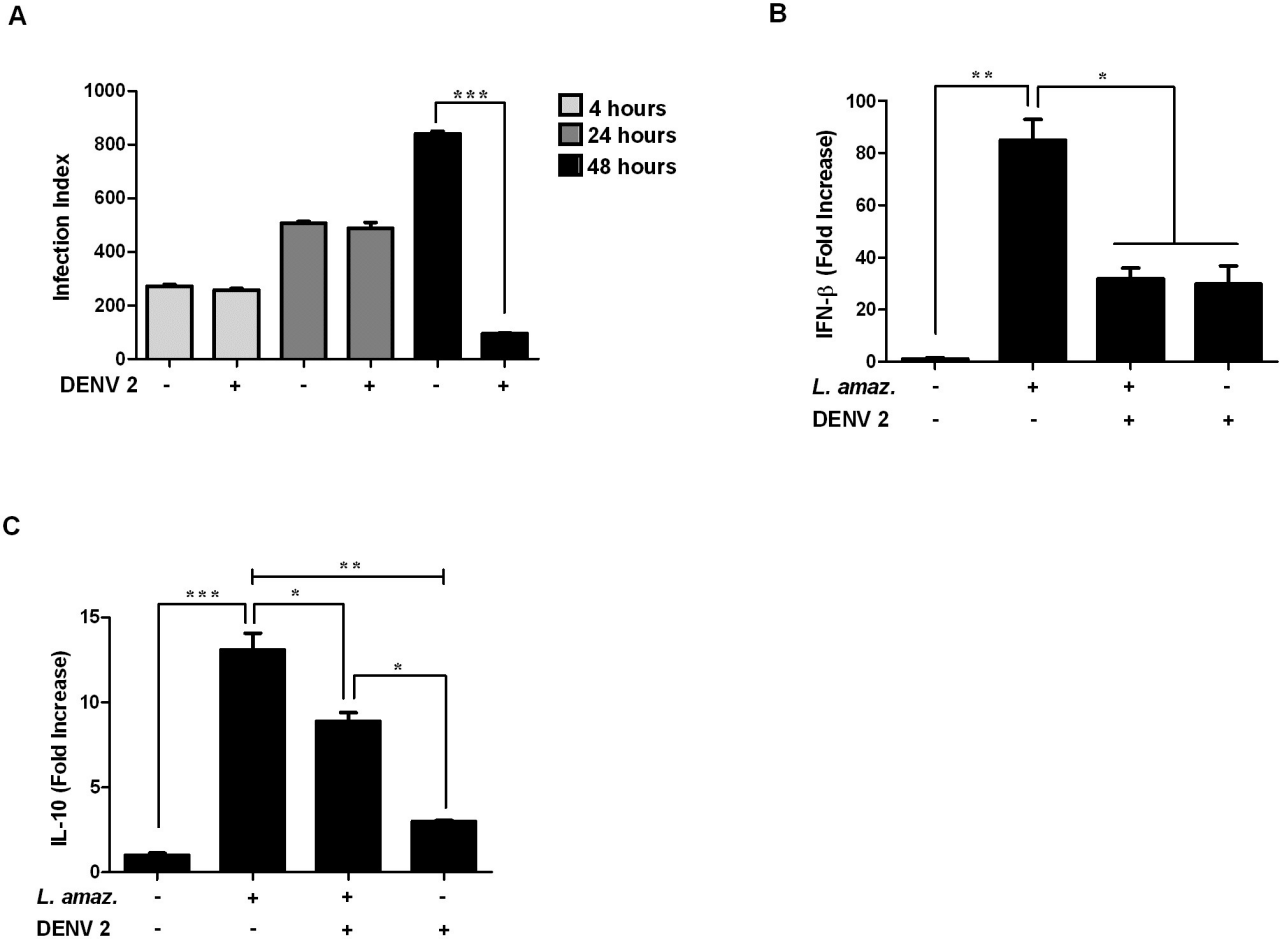
Dengue virus type 2 negatively modulates the intracellular growth of *Leishmania (L*.*) amazonensis* and prevents the expression of IFNβ and IL-10 in macrophages. Peritoneal macrophages from wild-type C57BL/6 mice were infected with Dengue virus-2 at a MOI of 2 for 1 h, followed by infection with stationary-phase promastigotes of *L*. *(L*.*) amazonensis* at a ratio of 5 parasites/cell for 48 h. (A) One hundred Giemsa-stained cells were inspected, and the infection index was calculated or after 4 h, total RNA was isolated and analyzed by quantitative real-time PCR for IFNβ (B) or IL-10 (C). Asterisks indicate significant differences between groups by ANOVA, with * *p* <0.05; ** *p* <0.0017; *** *p* <0.0001 (n = 3).

We aimed to address the question of whether other arboviruses widely transmitted in Brazil would also enhance the infection of *L*. *(L*.*) amazonensis*. To address this question, we used DENV2 in our experiments because its transmission may overlap with areas of cutaneous leishmaniasis. Thus, we measured the infection index to verify whether DENV2 could favor the entry, survival and/or intracellular growth of *L*. *(L*.*) amazonensis* (Fig 6A). We did not observe differences in the entry or survival index, 4 h or 24 h, respectively, of the parasites in DENV2 coinfection. However, 48 h postinfection, surprisingly, we observed the opposite effect observed with ICOV-*L*. *(L*.*) amazonensis* coinfection. Intracellular proliferation was significantly reduced in macrophages infected with DENV2, indicating that coinfected macrophages had a greater ability to reduce *L*. *(L*.*) amazonensis* infection.

Next, we evaluated the expression of IFNβ and IL-10 in DENV2-*L*. *(L*.*) amazonensis* coinfection, (shown in Fig 6B and 6C, respectively). We observed that in coinfected macrophages, the expression of both cytokines was reduced when compared to macrophages infected only with *L*. *(L*.*) amazonensis*. These data indicate that DENV2 may alter the expression profile of IFNβ and IL-10 induced in response to *Leishmania* infection, which may explain the reduction in the observed parasite load. These results may suggest specificity of the synergistic effect on parasite infection by phleboviruses, which share the *Leishmania* natural biological cycle.

## Discussion

Host-parasite interactions are modulated by a number of factors, including viral infections [35] that may shape the severity and the course of parasitic infections [36]. Parasite vectors host a plethora of organisms that may influence parasite transmission and life cycle [37]. Cutaneous leishmaniasis is highly prevalent in the Amazon region in Brazil [38, 39], which is an area of high transmission of arboviruses. Coinfections between *Leishmania* and phleboviruses may occur after the leishmaniasis vector insect bite. It has recently been demonstrated that TOSV increases both parasite burden and lesion size in a murine model of *L*.*(V*.*) guyanensis* infection [12], while *Leishmania* and *Phlebovirus* coinfection in sandflies have been reported [40, 41].

Phleboviruses are divided into genetically distinct groups: (i) Sandfly- or mosquito-borne phleboviruses: RVFV, Nápoles, Sicilian, Joa, Salobo, Candiru, Punta Toro, Salehabad and Aguacate or (ii) Tick-borne phleboviruses: Uukuniemi group, Bhanja group, Kaisodi group and Heartland group [41]. All phleboviruses are morphologically similar; they are 80–120 nm in diameter, have icosahedral symmetry, are enveloped and have two surface glycoproteins (Gn and Gc) [42]. They contain three single-stranded RNA segments (ssRNA)–S (small), M (medium) and L (large)—packaged within ribonucleoprotein particles (RNPs) by the N protein associated with an RNA-dependent RNA polymerase (RdRp). The S segment uses an ambisense coding strategy and codes for the N and NSs proteins. The NSs protein, likely the most important virulence factor, can block the production of type I IFN [34]. The M segment has negative polarity and encodes the Gn and Gc surface glycoprotein precursor, a 78 KDa structural protein. The NSm protein, another virulence factor with anti-apoptotic activity [43], also has negative polarity, and the L segment encodes RdRp [44].

We tested the hypothesis that *Phlebovirus* isolates from the Amazon region enhance *L*. *(L*.*) amazonensis* infection via the engagement of the RNA sensor PKR and the expression of type I IFN and IL-10. The Icoaraci and Pacuí viruses were isolated from *Nectomys squamipes* and *Oryzomys* sp., respectively, which are reservoirs of *L*. *(L*.*) amazonensis*, and were found in the same environmental as the vector *Lutzomyia flaviscutellata*. Our data confirm previous observations of the interaction between exogenous and endosymbiotic viral elements and the effects on the severity of cutaneous leishmaniasis [6, 8, 12]. However, we successfully investigated a putative *bona fide* model comprising a *Phlebovirus* isolated from a *L*. *(L*.*) amazonensis* sylvatic reservoir, which may resemble in part the natural cotransmission context. Moreover, we provide important novel observations regarding infection by ICOV and the interaction with *L*. *(L*.*) amazonensis in vitro* and *in vivo*.

Our data clearly demonstrated the role of the PKR/IFN1 axis in the enhancement of parasite infection in coinfected macrophages. PKR activation and IFNβ expression were observed due to ICOV infection and probably synergized with the induction by *L*. *(L*.*) amazonensis* infection. Thus, Icoaraci infection did not direct PKR degradation or suppression of IFNβ expression, as reported in other *Phlebovirus* infections [34, 45, 46]. The NSs protein encoded by some *Phlebovirus* species abrogates type I Interferon expression and promotes PKR degradation [47, 48]. However, preliminary data show that ICOV NSs sequences may be quite divergent when aligned to TOSV NSs sequences, and it is conceivable that they may exhibit other biological properties. Ongoing ICOV sequencing may reveal unknown features of New World *Phlebovirus* NSs proteins.

The viability of macrophages during ICOV infection or ICOV/*L*. *(L*.*) amazonensis* coinfection was reduced and this result was corroborated by the finding that ICOV does not activate Akt1, which is involved in cell survival. This feature distinguishes our results from the findings with *Leishmania (Viannia)* species harboring the symbiont LRV1, which promotes Akt activation and prolonged survival of infected macrophages [8]. We have not investigated whether Icoaraci infection would promote autophagy or apoptosis in macrophages and if such events would be related to reduction of macrophage viability. However, in our model, the number of amastigotes/infected macrophages is high, and further studies are necessary to unveil the signaling mechanisms in the coinfection model.

Our *in vivo* studies corroborated the *in vitro* observations and underline the importance of *Phlebovirus* and of the increase of IFNβ expression in aggravation of *in vivo Leishmania* infection. Large lesion size and increased parasite burden were observed in ICOV-*L*. *(L*.*) amazonensis* coinfected mice lesions. Importantly, the number of IFNβ-expressing cells was higher compared to *L*. *(L*.*) amazonensis* single infection. The inflammatory infiltrate was increased in the coinfection model and may indicate an exacerbation of the infection. It is conceivable that high type I IFN expression may tailor the pathogenesis as described by others [49]. Importantly, iNOS expression was not increased in ICOV/*L*. *(L*.*) amazonensis* coinfected lesion tissue, that may favor the parasite survival and replication.

One important finding from our work is that DENV2, also an RNA virus, does not promote *Leishmania* intracellular burden in *L*. *(L*.*) amazonensis*-DENV2 coinfected macrophages. We observed that in coinfected macrophages the expression of the transcripts of both cytokines was reduced when compared to the macrophages infected only with *L*. *(L*.*) amazonensis*. These data indicate that DENV2 is able to prevent the expression of IFNβ and IL-10 induced in response to *Leishmania* infection, which would justify the reduction in *L*. *(L*.*) amazonensis* load. Accordingly, recent studies have shown that DENV2 may impair Type 1 IFN signaling cascade in vertebrate cells leading to defective IFN expression [50, 51]. Expression of IL-10 and IFNβ in our model was evaluated 4 h postinfection, indicating that the reducing effect on the cytokines was caused in response to the virus, since according to data obtained in the proliferation assays (Fig 6A), the input of the parasites was not affected. This finding suggests a possible specificity of an exacerbation effect in *L*. *(L*.*) amazonensis* infection because *Phlebovirus* share and have coevolved with *L*. *(L*.*) amazonensis* in vectors and/or sylvatic reservoirs.

Considering the close relationship among phleboviruses, especially the Amazonian *Phlebovirus* serocomplexes that are often vectored by phlebotomine sandflies, one can speculate that it is possible that other phleboviruses, particularly members of Candiru group that infect humans [49], can be found coinfecting sandflies together with *Leishmania* [23], and obviously, that natural transmission of both virus and parasite is possible. These observations delineate a new scenario in which viral-parasite coinfection may aggravate *Leishmania* infection in humans and may contribute to the severity of clinical cases.

In conclusion, our work provides seminal evidence regarding novel molecular mechanisms involving *Phlebovirus-Leishmania* coinfection and may contribute to the understanding of CL pathogenesis.

## Supporting information

S1 Fig Icoaraci virus is able to infect and replicate in murine macrophages. (A) Peritoneal macrophages from wild-type C57BL/6 mice were coinfected with stationary-phase promastigotes of *L*. *(L*.*) amazonensis* at a ratio of 5 parasites/cell, Icoaraci virus or coinfected with both for 4 h, 24 h or 48 h. Total RNA was extracted and analyzed by semiquantitative PCR for the Icoaraci N nucleoprotein. (B) The supernatant of macrophages infected for 48 h was collected and titrated in a BHK-21 monolayer. After 3 days, the cells were fixed and stained with crystal violet.
(TIF)

S2 Fig Coinfection with Icoaraci virus potentiates BMDM infection by *Leishmania (L*.*) amazonensis*. (A) Bone marrow derived macrophages from wild-type C57BL/6 mice were infected with Icoaraci (BeAN 24262) virus for 1 h, followed by infection with stationary-phase promastigotes of *L*. *(L*.*) amazonensis* at a ratio of 5 parasites/cell. At 48 h postinfection, one hundred Giemsa-stained cells were inspected, and the infection index was calculated (percentage of infected macrophages multiplied by average number of amastigotes per macrophage). (B) The percentages of infected macrophages and (C) the number of parasites/100 cells were evaluated. (D) qPCR analysis for IFN1β expression was performed with the RNA obtained 4 h after Icoaraci- or Icoaraci/*L*. *(L*.*) amazonensis-*infection of BMDM C57BL/6 mice. Asterisks indicate significant differences between groups by Student’s *t*-test or ANOVA, with * *p* <0.0111; *** *p* <0.0001.
(TIF)
