# Supplementary figures and images for: Amazonian Phlebovirus (Bunyaviridae) potentiates the infection of Leishmania (Leishmania) amazonensis: Role of the PKR/IFN1/IL-10 axis
Source: PLoS Negl Trop Dis. 2019 Jun 19;13(6):e0007500. doi: 10.1371/journal.pntd.0007500 (PMC6602282; doi:10.1371/journal.pntd.0007500)

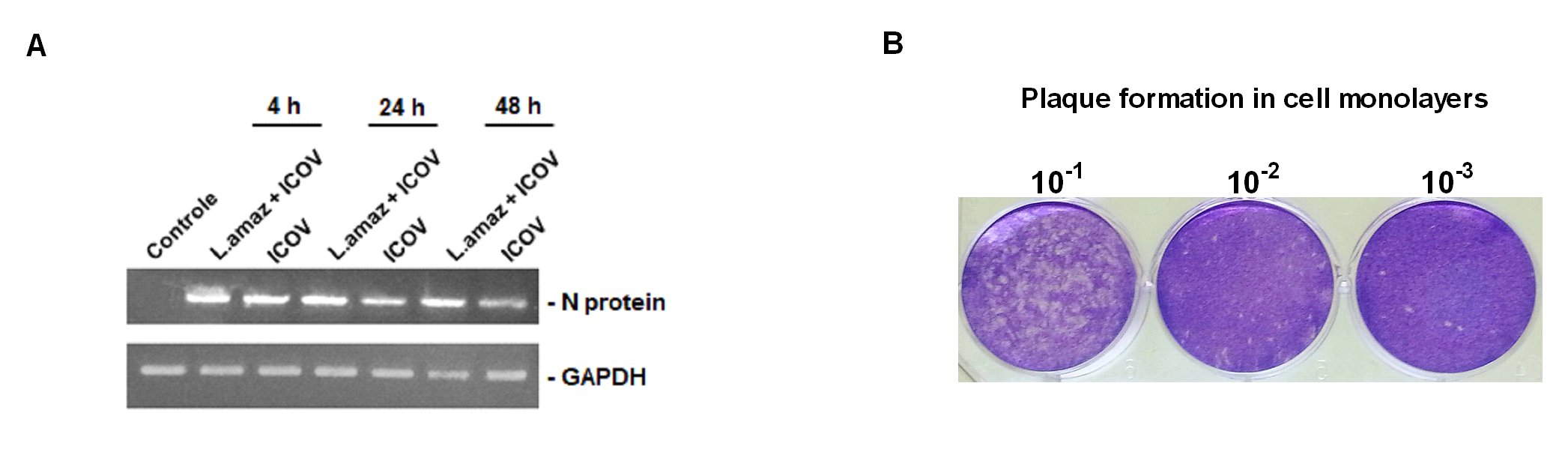

Supplement: S1 Fig — (A) Peritoneal macrophages from wild-type C57BL/6 mice were coinfected with stationary-phase promastigotes of L. (L.) amazonensis at a ratio of 5 parasites/cell, Icoaraci virus or coinfected with both for 4 h, 24 h or 48 h. Total RNA was extracted and analyzed by semiquantitative PCR for the Icoaraci N nucleoprotein. (B) The supernatant of macrophages infected for 48 h was collected and titrated in a BHK-21 monolayer. After 3 days, the cells were fixed and stained with crystal violet. (TIF) [file pntd.0007500.s001.tif]

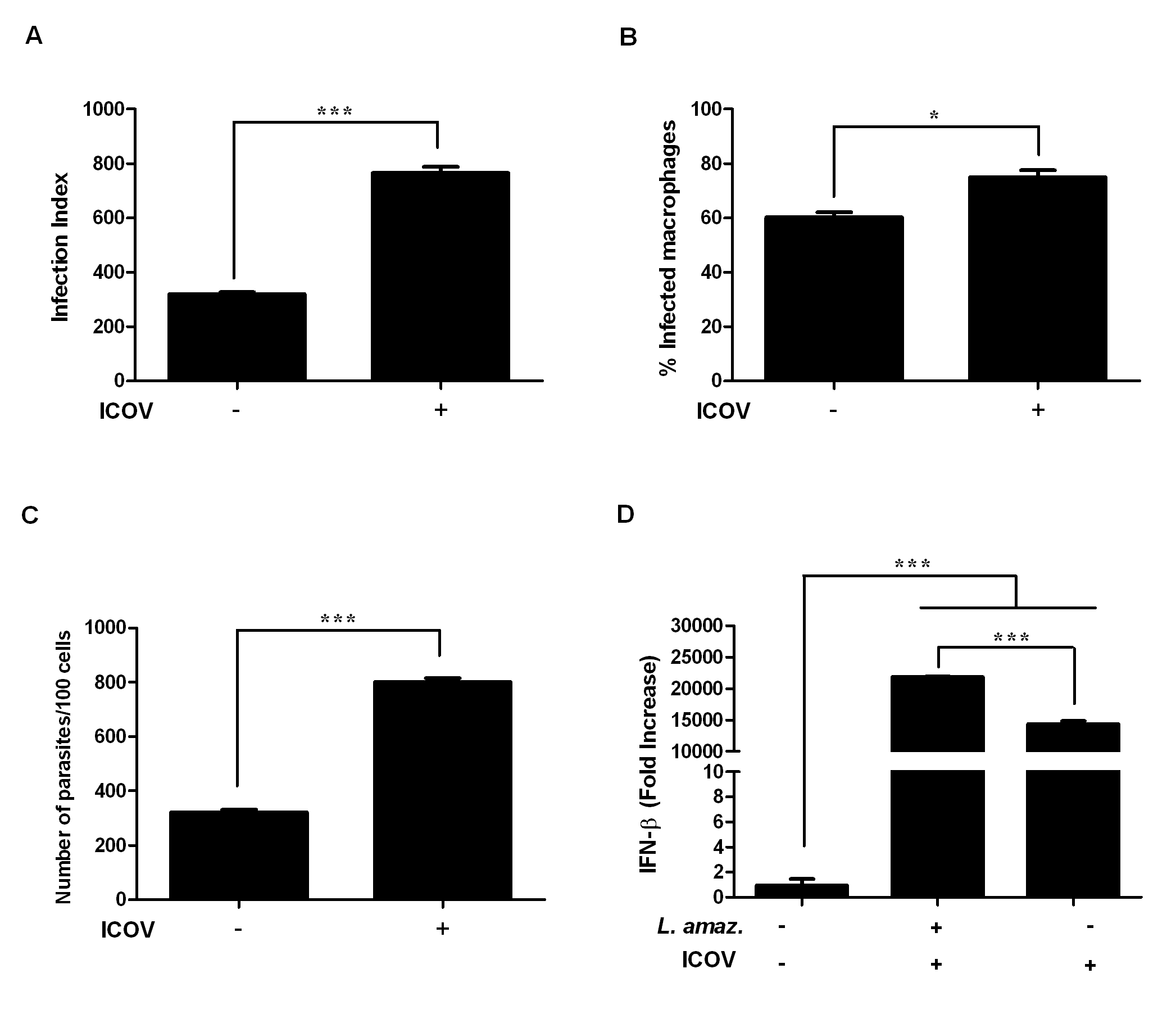

Supplement: S2 Fig — (A) Bone marrow derived macrophages from wild-type C57BL/6 mice were infected with Icoaraci (BeAN 24262) virus for 1 h, followed by infection with stationary-phase promastigotes of L. (L.) amazonensis at a ratio of 5 parasites/cell. At 48 h postinfection, one hundred Giemsa-stained cells were inspected, and the infection index was calculated (percentage of infected macrophages multiplied by average number of amastigotes per macrophage). (B) The percentages of infected macrophages and (C) the number of parasites/100 cells were evaluated. (D) qPCR analysis for IFN1β expression was performed with the RNA obtained 4 h after Icoaraci- or Icoaraci/L. (L.) amazonensis-infection of BMDM C57BL/6 mice. Asterisks indicate significant differences between groups by Student’s t-test or ANOVA, with * p <0.0111; *** p <0.0001. (TIF) [file pntd.0007500.s002.tif]
